# P-91. Clinical characteristics and analysis of predictors of the outcome of septic arthritis of the shoulder : a 16-year experience

**DOI:** 10.1093/ofid/ofae631.298

**Published:** 2025-01-29

**Authors:** Jae Eun Seong, Sangmin Ahn, Min Han, Yongseop Lee, Jung Ah Lee, Jung Ho Kim, Jin Young Ahn, Su Jin Jeong, Nam Su Ku, Jun Yong Choi, Joon-sup Yeom

**Affiliations:** Division of Infectious Diseases, Department of Internal Medicine and AIDS Research Institute, Yonsei University College of Medicine, Seoul, Seoul-t'ukpyolsi, Republic of Korea; Yonsei University College of Medicine, seoul, Seoul-t'ukpyolsi, Republic of Korea; Yonsei University School of Medicine, Seoul, Seoul-t'ukpyolsi, Republic of Korea; Division of Infectious Diseases, Department of Internal Medicine and AIDS Research Institute, Yonsei University College of Medicine, Seoul, Seoul-t'ukpyolsi, Republic of Korea; Yonsei University College of Medicine, seoul, Seoul-t'ukpyolsi, Republic of Korea; Yonsei University College of Medicine, seoul, Seoul-t'ukpyolsi, Republic of Korea; Yonsei University College of Medicine, seoul, Seoul-t'ukpyolsi, Republic of Korea; Yonsei University College of Medicine, seoul, Seoul-t'ukpyolsi, Republic of Korea; Division of Infectious Diseases, Department of Internal Medicine, Yonsei University College of Medicine, Seoul, Seoul-t'ukpyolsi, Republic of Korea; Yonsei University College of Medicine, seoul, Seoul-t'ukpyolsi, Republic of Korea; Division of Infectious Diseases, Department of Internal Medicine, Yonsei University College of Medicine, Seoul, Seoul-t'ukpyolsi, Republic of Korea

## Abstract

**Background:**

The number of septic arthritis cases is increasing due to the increasing number of joint surgeries and lengthened life expectancy. There are few data about the characteristics and risk factors of septic arthritis of the shoulder. As timely diagnosis and treatment are essential, we aim to describe the clinical characteristics and analyze predictors of the outcome of Septic arthritis of the shoulder.

Microorganisms causing septic arthritis of the shoulder

**Figure:**
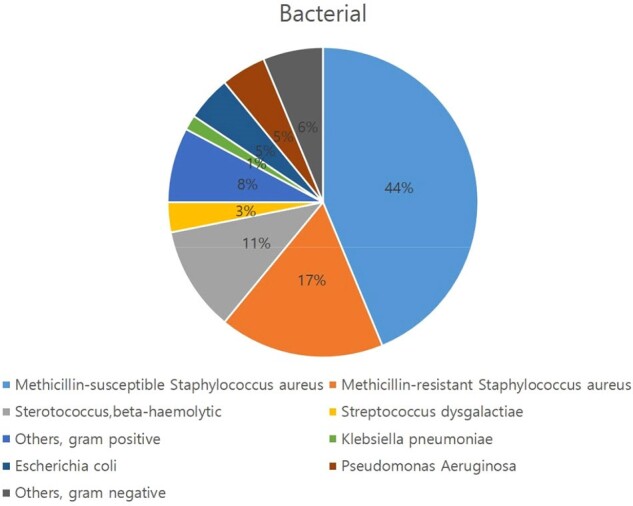

**Methods:**

We performed a retrospective cohort study of patients diagnosed with septic arthritis of the shoulder between June 2007 and June 2023 at a tertiary hospital in Korea, Aged over 19. We excluded patients who did not undergo treatment in this hospital and had tuberculosis isolated from synovial culture.

We analyzed demographic data, comorbidities and underlying conditions, shoulder trauma history, operation history, acupuncture history, previous steroid injection history, rotator cuff tear, clinical presentation, MRI finding, synovial findings, laboratory findings, and culture findings. We analyzed whether each characteristic was associated with unfavorable outcome.

**Figure:**
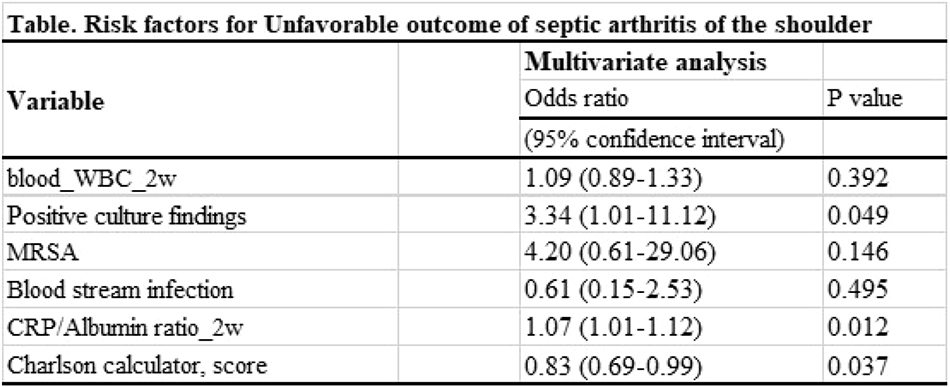

**Results:**

Among 99 patients enrolled in this study, 49 patients showed unfavorable prognosis.

The most frequently identified microorganisms were *Staphylococcus aureus* (39 cases), 11 of which were Methicillin-resistant *S. aureus* (MRSA), *Streptococcus*, and *beta-haemolytic* (7 cases).

The univariate analysis revealed that elevated blood WBC count at 2 weeks and CRP/Albumin ratio at 2 weeks were significant factors. Additionally, findings of positive culture, blood stream infection and MRSA isolation were statistically significant.

In multivariate analysis, we identified positive culture findings (OR 3.34; 95% CI, 1.01 – 11.12; P = 0.049), the CRP/albumin ratio at 2 weeks (OR 1.07; 95% CI, 1.01 – 1.12; P = 0.012), and the Charlson calculator score (OR 0.32; 95% CI, 0.69 – 0.99; P = 0.037) as risk factors for unfavorable outcomes.

**Conclusion:**

The significance of our study is that we analyzed characteristics of multiple cases for the long term and identified new predictors of the outcome for septic arthritis of the shoulder. In case of identified positive culture findings, elevated CRP/albumin ratio at 2 weeks and high Charlson calculator score, unfavorable outcome can be considered.

**Disclosures:**

**All Authors**: No reported disclosures

